# Some Account of a New Theory of Galvanic Electricity, Founded on Experiments

**Published:** 1807-09

**Authors:** M. J. A. Heidman

**Affiliations:** Physician in Vienna


### *Some Account of a new Theory of* GALVANIC ELECTRICITY, *founded on Experiments.* By M. J. A. HEIDMAN, Physician in Vienna.

M. HEIDMAN sets out with giving a succinct account of Galvanic Electricity, relatively to the discovery of the Voltaic pile. He includes under the same head, the Observations of Galvani on animal electricity; the Experiments of Valli; the Essays of Volta, in opposition to the advocates of a particular animal electricity; the Dissertations of Fontana, and of Professor Reil; Du Creve's Essay on the irritability excited in a vacuum; the Inquiries of Fowler, Pfaff, Humboldt and Ritter; and lastly, he notices the experiments which led Volta to the discovery of the electrical pile, which M. Heidman designates by the appellation of *Galvanic Battery*. He next proceeds to give a description of this apparatus, of the several parts of which it is composed, and of their different dispositions. In this part of the work he treats successively of the simple galvanic chain; of the nature of conductors; of their mass and extent; of the properties of solid or liquid conductors; and of the qualities attributed to solid and moistened conductors.

M. Heidman next considers the experiments and opinions of the various authors who have written on this subject: after which he proceeds to explain his own theory, which he supports by appropriate observations.

Two experiments which he made with a view of determining the influence of humid bodies in the galvanic chain are related. The first consists in placing in contact two homogeneous metals with the nerves and muscles of a frog, and establishing a communication between the two metals by means of zinc and silver; in this case no contraction is perceived, either in closing or opening the chain, which is not in conformity with the opinion of Volta; there appears then to be wanting the essential condition, viz. *humidity*, between the two heterogeneous metals.

In the second experiment he plunges the metallic plate into vessels containing salt water, and the electricity becomes augmented on increasing the quantity of the liquid, so as to bring it in contact with a greater surface of the metals. The author here refers to similar experiments by Messrs. Desormes and Van Marum, who also concluded that the size of the moist surfaces greatly contributed to the strength of the pile.

M. Heidman afterwards endeavours to ascertain the power of the different fluid conductors, according to the degree of their chemical action. For example, having placed two prepared frogs in such a position, that their nerves were inserted in watch glasses containing different liquids, such as a solution of alkaline sulphuret on one side and water on the other, and the communication of the two fluids being established by means of a metallic wire, he observed, that at the instant of the formation of the chain it was the muscle on the side of the first of these liquors that became contracted, and that immediately on the rupture of the chain, the muscle on the side of the water alone suffered the contraction.

In order that the stimulant qualities peculiar to some liquors, and particularly to the acids, might not lead him into error, he interposed between the nerve and the liquor, a piece of flesh well soaked in water.

In this manner he submitted to experiment all the different liquids already known as conductors of ordinary electricity, and he has arranged them in the following order, relative to their chemical power, and their galvanic action.

Acids—oxymuriatic.
—— muriatic, impregnated with azote.
—— muriatic.
Solution of oxymuriate of pot-ash.
Acids—sulphuric.
—— fluoric.
Sulphurets—alkaline.
—— earthy.
Acids—phosphoric.
—— arsenic.
—— oxalic.
—— boracic.
—— molybdic.
—— acetic.
—— benzoic.
—— gallic.
Solution of ammonia.
—— pot-ash.
—— soda.
—— phosphate of soda.
—— ammoniacal acetite
—— ammoniacal muriate.
Solution of martial muriate.
—— ammoniacal nitrate
—— ammoniacal sulphate.
—— muriate of pot-ash.
—— nitrate of pot-ash.
—— sulphate of pot-ash.
—— phosphate of pot-ash.
—— acetite of pot ash.
—— muriate of soda.
—— nitrate of soda.
—— sulphate of soda.
—— phosphate of soda.
—— acetite of soda.
—— muriate of barytes.
—— muriate of lime.
—— muriate of magnesia.
—— sulphate of alumine
—— nitrate of silver.
—— sulphate of copper.
—— sulphate of iron.
—— sulphate of zinc.

Solution

Solution of acetite of lead.
Citron juice.
Solution of acidulated tartrite of pot-ash.
——— tartrite of pot-ash.
——— emetic tartar.
Water saturated with carbonic acid gas.
Solution of ammoniacal carbonate.
——— carbonate of potash.
——— carbonate of soda.
Lime water.
Recent urine.
Serum of the blood.
Blood.
White of eggs.
Fresh muscles.
Nerves, while yet moist.
Water.
Sweetened water.
Saliva.
Juice of fresh plants.
Milk.
Wine.
Unrectified alcohol.
Rectified alcohol does not act as a conductor.

The author draws the following conclusions from his experiments on the above liquids.

The oxydating liquid, first in the order, possesses the greatest chemical power, and determines the oxygen pole with the greatest number of the solid conductors.

The liquid of an inferior rank is therefore only a simple conductor, which in the apparatus for the decomposition of water, indicates the hydrogen pole. The oxydating, or irritating liquid, and the liquid conductor, ought therefore to be distinguished from each other. He observes, however, that the order he has assigned to these liquids, is not constant and uniform, unless they be employed at an equal degree of concentration.

M. Heidman afterwards examines the action exercised by these liquids upon the various solid conductors, which he regards as depending upon chemical affinity, and which is manifested by the displacement of the poles, in the same manner as with zinc; for instance, the acids precede the sulphurets and determine the oxygen pole, as with lead, tin, &c.; while with gold and platina, the alkaline and earthy sulphurets have a superiority even over the acids, and leave to them the hydrogen pole only.

In treating of the galvanic chain, the author makes two distinct classes.

The first he represents by the following series:

*Metal more oxydizable — water — metal less oxydizable* than the body communicating by immediate or intermediate contact with the two solid conductors. In this chain the oxygen pole is always according to the direction in which the two solid heterogeneous bodies touch the water.

 The

The second is represented by this series, *oxydizing liquid—solid oxydizable conductor—liquid simply a conductor;* then follows the communication of the two heterogeneous liquids.

Thus in a chain formed of *silver—water—zinc—silver—water—zinc;* the first plate of silver and the last of zinc are, according to this author, superfluous conductors, and ought not to be considered as essential parts of the battery, since by these two plates the chain is only compleated, and the two chains of six members are changed into a battery of two. Consequently, when the pile is formed with plates of copper and zinc soldered, it should be terminated at the two extremities by simple plates, in order to avoid superfluous conductors, since it is not by placing the copper or zinc at one of these extremities which decides the hydrogen and the oxygen pole, but only the respective disposition of the two heterogeneous metals, and their contact with water; whence he concludes, that the plates by which the pile is terminated, are superfluous members of the battery, in opposition to the opinion of Volta, Carlisle, and all those who assert that the galvanic action is produced only by the contact of two heterogenous conductors, whether liquid or solid.

In the third division of the work, a detail is given of the phenomena exhibited spontaneously, and without any foreign aid, by the battery of Volta, when in a state of the greatest activity: the author enumerates seven principal ones.

1st. The peculiar odour perceived after a short time, differing a little according to the nature of the conductors, which are for the most part metallic.

2d. The slight noise or crackling produced by a strong battery, whether in the pile or in the dish apparatus, and which proceeds from the hydrogen gas, liberated sometimes even with a sort of white froth.

3d. The changes which the moistened conductors undeigo in both kinds of apparatus, such as the decomposition of water, the saline efflorescences, the presence of a free alkali, when neutral salts are employed, &c. &c. A more accurate investigation into the nature of these changes is regarded by M. Heidman as opening a vast field for new experiments.

4th. The oxydation of the metals, which the present author regards, in common with many others, as essential to galvanic action. It occurs, he observes, when the chain is formed, not only in atmospheric air, in nitrous and and oxygen gases, in oxyde of azote, in acid gases, but also, though in a less degree, in the vacuum of the air pump, and in those gases which contain no oxygen, such as hydrogen and azotic gas. He discovers in these circumstances what principally constitutes the difference between the battery of Volta and the common electrical machine, in which no electricity is produced without the presence of oxygen. In the galvanic battery, oxygen is furnished by the water, by the liquors in which it is contained, and by the acids, and its action is powerful in proportion to the abundance of this principle.

5th. The absorption of oxygen. One of the most remarkable experiments is, that in which Professor Schaul observed the decomposition of common air under the bell-glass; the cessation of action when the oxygen becomes exhausted, the renewal of the action even with commotion, on furnishing a supply of fresh air, are phenomena which prove, in the opinion of M. Heidman, that the presence of oxygen is necessary to produce this action, and that it depends on a chemical operation.

6th. The immediate action of the Voltaic battery upon Bennet's electrometer. When this instrument is brought near to the hydrogen pole, the divergency of the gold leaves is stronger than when it is brought near the oxygen pole. Immediate contact is not necessary to produce this effect; it is sufficient that the base of the electrometer communicates with the earth, to establish a conductor so as to produce a divergency; which is, however, in this case more feeble. The author was not able to ascertain two opposite states of electricity in the hydrogen and oxygen poles. He employed with this intention a very strong Voltaic battery, and an electrometer formed of a very narrow and sensible glass cylinder; on establishing, by means of a conductor, the communication between the hydrogen pole and the top of the instrument, the divergency nearly carried the gold leaves to the sides; and they remained in the same situation when he established the communication with the oxygen pole, which would be impossible in the case of two contrary electricities. If a communication be formed by means of a good metallic conductor, between the Voltaic battery and a prepared frog, which does not communicate with the ground, no movement whatever takes place; which affords a convincing proof, that in every galvanic action there must be in reality, a discharge, or partition of the galvanic electricity, which did not occur in the present experiment.

 7th.

7th. This article has for its object the spark of the Voltaic battery, as observed in the dark by Nicholson. Here the author also relates the observations of Pfaff, Hebebrandt, Biot, and Halle, by which it appears, that independently of the closing of the chain by the two extremities of the battery, a light often appears on the pile itself, that is, on the sides of the metallic plates. M. Heidman thinks himself warranted in doubting this phenomenon, the observation of which appears to him not to have been accurate, or to be reconcilable with received principles. He supposes, that in the dark we may close, by means of the conductor, only a part of the battery from which the spark has been elicited.

From the above analysis, it must be evident, that M. Heidman frequently differs from those whose opinions he details, and that he builds his own doctrines on experiments peculiar to himself.

